# Association of gut microbiota and short-chain fatty acids with pre-diabetes and diabetes following gestational diabetes

**DOI:** 10.3389/fmicb.2026.1681153

**Published:** 2026-01-26

**Authors:** Beibei Gao, Qiong Shen, Jingyu Yang, Ying Wu, Dandan Hu, Lei Chen

**Affiliations:** Department of Endocrinology, Suzhou Municipal Hospital, The Affiliated Suzhou Hospital of Nanjing Medical University, Suzhou, China

**Keywords:** gestational diabetes mellitus, glucose metabolism disorders, gut microbiota, pre-diabetes, short-chain fatty acids

## Abstract

**Background:**

Emerging evidence demonstrates correlations among the gut microbiota, fecal short-chain fatty acids (SCFAs) and glucose metabolism. Few studies focus on post-gestational diabetes women. This study aimed to compare the gut microbiota and fecal SCFAs among different states of postpartum glucose metabolism in women with previous gestational diabetes mellitus (p-GDM).

**Methods:**

The study finally recruited 60 women with p-GDM including 16 healthy controls (HC), 40 Pre-diabetes (Pre-DM) patients and 4 type 2 diabetes patients according to a 2-h 75-g oral glucose tolerance. Stool samples were obtained 1–5 years after delivery. Gut microbiota was obtained by sequencing V3–V4 region of 16S rRNA gene and fecal SCFAs were measured by gas chromatography-mass spectrometry. The microbial community structure of the Pre-DM group, as revealed by principal coordinates analysis (PCoA), exhibited distinct clustering that was further validated by hierarchical clustering, definitively identifying two subgroups: Pre-DM1 and Pre-DM2. The primary analyses in this report compared the HC and Pre-DM groups, with a particular focus on a three-group comparison (HC vs. Pre-DM1 vs. Pre-DM2) to demonstrate heterogeneity within Pre-DM. For the type 2 diabetes group, only descriptive statistics were presented, without formal statistical testing.

**Results:**

No significant differences were observed in age, BMI and months post-delivery between HC and Pre-DM group. The Pre-DM group exhibited two distinct clustering patterns: Pre-DM1 (*n* = 25) and Pre-DM2 (*n* = 15). The gut microbiota structure of Pre-DM1 largely overlapped with the HC group, while Pre-DM2 was closer to the type 2 diabetes group. Compared to the HC group, the relative abundance of *Faecalibacterium, Ruminococcus*, and *Subdoligranulum* remained unchanged in Pre-DM1 group, while significantly reduced in Pre-DM2 group. Furthermore, compared with HC group, acetic acid and propionic acid were increased in the Pre-DM1 group while were similar to Pre-DM2. HC group had higher concentration of caproic acid than Pre-DM1 (*P* = 0.01) and Pre-DM2 (*P* = 0.02).

**Conclusions:**

Our study discovered that dysbiosis of the gut microbial structure and alterations in SCFAs had already been present in women with Pre-DM and further revealed two subsets of Pre-DM with remarkable heterogeneity. Further studies are needed to explore whether the heterogeneity can help predict postpartum glycemic states.

## Introduction

Gestational diabetes mellitus (GDM) is defined as glucose intolerance that begins or is first diagnosed during pregnancy in the absence of pre-existing overt diabetes diagnosed (American Diabetes Association Professional Practice Committee, 2022). While the prevalence of GDM varies globally from 1% to over 30% (Lain and Catalano, 2007), an estimated prevalence of 15% was reported in Chinese pregnant women according to the criteria from the International Association of the Diabetes and Pregnancy Study Groups (IADPSG; Lauenborg et al., 2004). In the short term, GDM is associated with an increased risk of adverse pregnancy outcomes such as premature delivery, cesarean section, preeclampsia, fetal abnormalities, and intrauterine death (Xu et al., 2025). Moreover, a history of GDM predisposes women to a significantly increased long-term risk of developing type 2 diabetes, with 40% of affected women developing type 2 diabetes within a 10–15 years postpartum (Lynch and Pedersen, 2016). Although decreased insulin sensitivity and enhanced nutrient absorption are beneficial for normal pregnancy, may reveal a preexisting deficiency in insulin secretion and insulin sensitivity (McIntyre et al., 2019). Thus, the stress of pregnancy may reveal a predisposition to type 2 diabetes and provide early signs that are useful for preventing chronic diseases. Despite numerous investigations into the pathophysiology of GDM, the precise mechanisms remain incompletely understood, with gut microbiota emerging as a significant contributing factor.

Gut microbiota dysbiosis has been implicated in various diseases spanning gastroenterological, neurologic, respiratory, metabolic, hepatic, and cardiovascular illnesses (Zhu et al., 2013). The landmark study by Bäckhed and colleagues in 2004, which first established the vital role of gut microbiota in glucose metabolism, stimulated extensive research on this relationship (Bäckhed et al., 2004). Accumulating evidence from both human and animal studies suggests that insulin resistance is characterized by reduced alpha diversity and distinct shifts in beta diversity (Le Chatelier et al., 2013; Kubota-Takamori et al., 2025). This pattern of microbial alteration has also been observed in Women with GDM (Kuang et al., 2017). At the phylum level, a decreased ratio of *Bacteroidetes* to *Firmicutes* has been associated with both obesity and GDM (Ley et al., 2006; Cortez et al., 2019). At the genus level, increased abundances of *Parabacteroide*s (Kuang et al., 2017), *Fusobacterium, Prevotella* (Turnbaugh et al., 2006), *Blautia* (Crusell et al., 2018; Ye et al., 2019), *Collinsella* (Cortez et al., 2019; Crusell et al., 2018), *Desulfovibrio* (Crusell et al., 2018), and *Eubacterium_hallii_group* (Ye et al., 2019), alongside reduced abundances of *Faecalibacterium* (Turnbaugh et al., 2006; Crusell et al., 2018; Ye et al., 2019), *Roseburia* (Kuang et al., 2017; Ye et al., 2019), *Bacteroides* (Turnbaugh et al., 2006), *Clostridium* (Kuang et al., 2017), and *Eubacterium rectale* (Cortez et al., 2019) were observed in the women with GDM compared to those without GDM. Although the specific microbiome features identified in women with GDM are different, one consistent finding is that short-chain fatty acids (SCFAs)-producing genera significantly reduced in women with GDM (Kuang et al., 2017; Cortez et al., 2019; Turnbaugh et al., 2006; Crusell et al., 2018; Ye et al., 2019). SCFAs are the main metabolites produced by bacterial fermentation of indigestible carbohydrates in the gastrointestinal tract (Miller and Wolin, 1996). Growing evidence suggests that SCFAs have anti-obesity and anti-diabetic effects through pleiotropic mechanisms (Chambers et al., 2015; Zhao et al., 2018).

Studies on gut microbiota in women with previous GDM (p-GDM) are limited, even though nearly half of these individuals have persistent postpartum glucose intolerance (Hasain et al., 2020). Furthermore, existing studies primarily focus on comparing gut microbiota between women with and without p-GDM. To our knowledge, there is only one study from Malaysia comparing gut microbiota composition in p-GDM women with and without postpartum glucose intolerance. However, the study cohort was comprised predominantly of women with pre-pregnancy obesity. Moreover, the research did not distinguish type 2 diabetes from pre-diabetes and grouped both conditions under the term “glucose intolerance.”

Hence, we aimed to investigate the differences in the gut microbiota and fecal SCFA profiles across a spectrum of postpartum glucose metabolism states: healthy controls (HC), Pre-diabetes (Pre-DM) and type 2 diabetes in the women with p-GDM.

## Materials and methods

### Study population

**Figure 1:**
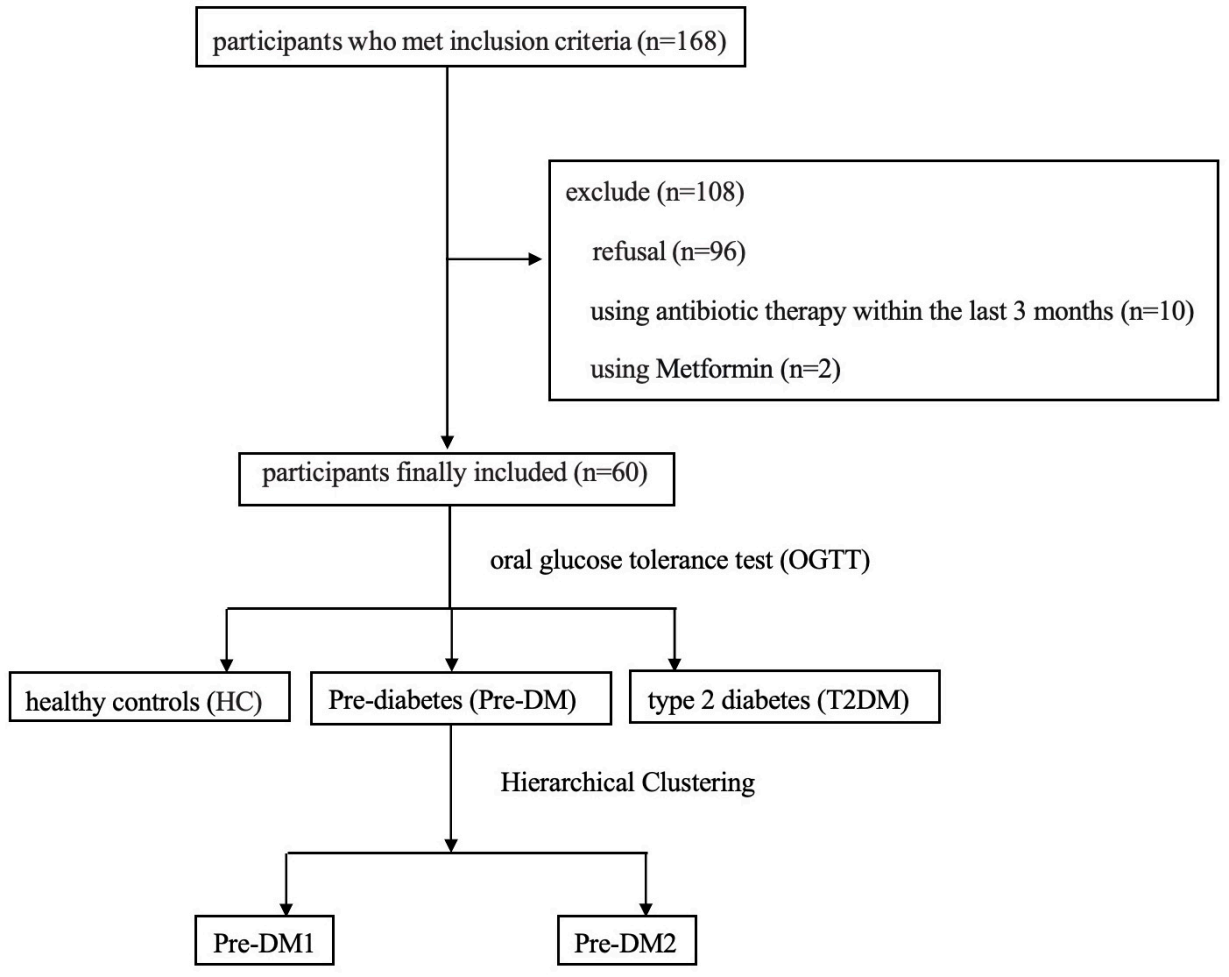
Flowchart and design of the study.

This study retrospectively recruited subjects from women diagnosed with GDM from 2017 to 2020 at the Maternity and Child Health Center of Suzhou Municipal Hospital. The diagnosis of GDM was based on the criteria of IADPSG (Lauenborg et al., 2004). A total of 168 women were briefly interviewed by telephone and were asked to attend a follow-up visit. Of these, 72 women completed this visit at 1–5 years postpartum. All subjects were of Han Chinese descent. Women with the following characteristics were excluded from the study: a history of antibiotic therapy within the last 3 months; chronic diseases requiring medication, except for levothyroxine; and a history of smoking or drinking. Finally, 60 women were enrolled in the study and underwent a 75-g OGTT. Based on the diagnostic standards of American Diabetes Association in 2010 (American Diabetes Association, 2010), participants were categorized into three groups: HC, Pre-DM, and type 2 diabetes (Figure 1). Written informed consent was obtained from all subjects before their participation. The study was conducted in accordance with the Declaration of Helsinki and was approved by the Ethics Committee of Suzhou Municipal Hospital (No. KL901540).

### Anthropometric and clinical assessments

The anthropometric data (weight, height, waist, and hip circumference) were measured, and lifestyle factors and medication history were obtained from clinical medical records suing a standard questionnaire. Waist was measured in an erect position at the mid-point between the iliac crest and the lower costal margin, and hip circumference at the widest portion of the buttocks, respectively. Systolic and diastolic blood pressure (expressed in mmHg) were measured twice (Omron Model HBP-1100, Omron Company, Dalian city, Liaoning Province, China) with the participant in a sitting position, and the mean value was used for further analysis.

Following an overnight fast of at least 8 h, the women were examined between 07:00 and 09:00. A standard 2-h OGTT with 75 g of glucose were performed and blood samples were drawn at 0, 0.5, 1, and 2 h. Serum samples were used to detect glucose and C-reactive protein (CRP) levels on a fully automatic biochemical analyzer (Hitachi 7000, Tokyo, Japan). Whole blood samples were used for the determination of HbA1c level by high-performance liquid chromatography (HLC-723G8, Tosoh Bioscience, Japan). Serum insulin was measured using a Human Insulin ELISA Kit (Sigma-Aldrich, St. Louis, MO, US). The area under the curve (AUC) for glucose and insulin was calculated following the trapezoidal rule. Glucose was expressed in mmol/l and insulin was expressed in μLU/mL. Body mass index (BMI) = weight/height^2^. To quantify insulin sensitivity and insulin function, the relevant index was calculated from the OGTT. Homeostasis model assessment of insulin resistance (HOMA-IR) = fasting insulin (INS) × fasting glucose/22.5; HOMA-β = 20 × fasting INS/(fasting glucose −3.5); peripheral glucose uptake rate (M) = 7,500/120 + (fasting glucose-2 h glucose) × 1.15 × 180 × 0.19 × weight/120; insulin sensitivity index (ISI) = M/{(fasting glucose + 2 h glucose)/2 × Ig [(fasting INS + 2 h INS)/2]}; Disposition index (DI) = I = ISI × AUC INS/AUC glucose (Stumvoll et al., 2000).

### Stool sample collection

Fecal samples were collected at home using sterile fecal collection tubes by the participants within 48 h after the OGTT. Samples were transferred to the lab within 6 h and then stored at −80 °C under a uniform protocol until analysis. A total of 60 fecal samples were used for 16S rRNA sequence. Among these, 52 samples were used for SCFAs and 8 samples were excluded due to insufficient sample volume.

### DNA extraction and 16S rRNA PCR

The total bacterial genomic DNA was extracted from frozen fecal samples using SPIN easy DNA kit (ZEPING Biotech, Beijing, China) following the manufacturer's instructions. The NanoDrop spectrophotometer (Thermo Fisher Scientific, Waltham, MA, USA) and agarose gel electrophoresis were used to determine the quality and quantity of extracted DNA. The V3–V4 hypervariable region of the bacterial 16S rRNA gene was amplified by PCR using barcoded universal primers (F: 5′-ACTCCTACGGGAGGCAGCA-3′, R: 5′-GGACTACHVGGGTWTCTAAT-3′). Sequencing libraries were prepared using the TruSeq Nano DNA LT Library Prep Kit from Illumina and Paired-end sequencing of the libraries was performed on a NovaSeq at Suzhou Bionovogene Co., Ltd. (Suzhou, China). The raw data was analyzed using the QIIME2. Sequence assembly, quality control, and clustering were then performed.

### Fecal short-chain fatty acids measurement

Fecal levels of SCFAs, including acetic acid, butyrate, isovaleric acid, caproic acid, isobutyric acid, valerate acid, and propionic acid were measured using gas chromatography-mass spectrometry (GC-MS, Agilent 7890A/5975C instrument, HP-5MS column, 0.25 × 30 mm, 0.25 μm particle size) at Suzhou Bionovogene Co., Ltd. The total SCFA levels were determined as the sum of these seven SCFAs. Briefly, 100 mg of the sample was weighed and mixed it with 1 mL of 0.005 M NaOH solution and 50 μL of 2-methylbutyric acid for 2 min, then was incubated at 4 °C for 2 h. The mixture was mixed for 2 min, and was centrifuged at 4 °C for 20 min at 13,000 rpm. A 500 μL of the supernatant was transferred to a clean tube, with 300 μL of distilled water and 500 μL of isopropanol/pyridine solution (3:2, v/v). After derivatization with platelet cytotoxin solution, we analyzed it using 500 μL of n-hexane extraction. Chromatographic separation was performed using an Agilent HP-5 capillary column. The injection ratio was 10:1, with an injection volume of 1 μL. The temperature of inlet, ion source, and transfer line were set at 280, 230, and 250 °C, respectively. The initial temperature for the programmed temperature rise was 60 °C, held for 5 min, and then increased to 250 °C at 10 °C/min. The helium carrier gas flow rate was 1.0 mL/min. Data processing was conducted using Agilent MSD ChemStation (E.02.00.493, Agilent Technologies, USA).

### Statistical analysis

Continuous data with normal distribution or approximately normal distribution were presented as the mean ± standard deviation (SD) for variables. Comparisons between two groups were performed using Student's *t*-test, while comparisons across more than two groups were analyzed using one-way analysis of variance (ANOVA) followed by *post-hoc* LSD test for in-between groups' comparisons. Categorical variables were presented as numbers (%) and group differences were assessed using Fisher's exact test. Given the limited number of participants (*n* = 4), type 2 diabetes group was excluded from formal comparative statistical analyses; only descriptive statistics were presented for this subgroup. Consequently, the primary analyses focused on comparisons between the HC and Pre-DM groups. Operational taxonomic unit (OTU) clustering and taxonomic classification were performed using QIIME2 (version 2019.4). Analysis of beta diversity using Bray-Curtis distances and PCoA visualization showed differential clustering of the Pre-DM group. To validate the observed grouping pattern, hierarchical clustering was performed on the OTU abundance data. This analysis identified two distinct sub-clusters within the cohort, designated as Pre-DM1 and Pre-DM2 (Figure 1). Permutational multivariate analysis of variance (PERMANOVA) confirmed a statistically significant difference in microbial community composition between Pre-DM1 and Pre-DM2 (*p* = 0.001), with the grouping factor explaining 9.47% of the variance (*R*^2^ = 0.0947). Furthermore, distance analysis revealed that the mean Bray-Curtis distance between the two groups (0.875) was greater than the mean distance within each group (0.783), supporting the existence of differences between groups. Linear discriminant analysis effect size (LEfSe) measurements were employed for further statistical analysis of gut microbial communities across the four groups, with a linear discriminant analysis (LDA) score > 2. The Microbiome Multivariable Associations with Linear Models (MaAsLin2) method was applied to control for potential confounding factors. Spearman correlation analysis was utilized to ascertain the relationships between the gut microbiota, SCFA levels, and clinical characteristics. The eight missing SCFA values were handled using the k-nearest neighbors (KNN) imputation method. Statistical analyses were performed using the Statistical Package for the Social Sciences version 21.0 software and the R software (version 4.3.0) with a *P* < 0.05 was considered statistically significant.

## Results

### Clinical characteristics of the study cohort

The characteristics of the subjects are summarized in Table 1. The participants were grouped to three groups based on the OGTT results: 16 women in the HC group, 40 in the Pre-DM group and 4 in the type 2 diabetes group. No significant differences were observed between the HC and Pre-DM groups in terms of age (36.19 ± 3.8 vs. 36.98 ± 4.76 years), BMI and months post-delivery. Other clinical factors, including education level, family history of diabetes and BP, were also similar between the two groups. As expected, markers of glucose homeostasis were higher in the Pre-DM group compared with the HC group (Table 1). Similarly, fasting insulin and HOMA-IR were elevated in Pre-DM group (both *P* < 0.01) while ISI was significantly lower.

**Table 1:** Characteristics of the study population.

**Characteristics**	**HC** **(*N* = 16)**	**Pre-DM** **(*N* = 40)**	**T2DM** **(*N* = 4)**	***P***
				**HC vs.** **Pre-DM**
**Descriptive measurements**				
Age, years	36.19 ± 3.8	36.90 ± 5.08	34.75 ± 6.08	
High school education and above, *n* (%)	14 (87.5%)	33 (82.5%)	2 (50%)	
Family history of diabetes, *n* (%)	7 (43.8%)	15 (37.5%)	2 (50%)	
Insulin requirement during pregnancy (%)	2 (12.5%)	19 (47.5%)	3 (75%)	0.017
Months post delivery	42.19 ± 15.41	37.03 ± 14.81	27.50 ± 19.28	
Weight, kg	61.76 ± 13.37	58.52 ± 9.42	71.98 ± 19.95	
BMI, kg/m^2^	24.14 ± 4.92	23.51 ± 3.03	28.23 ± 6.73	
Waist, cm	79.57 ± 11.14	82.07 ± 7.24	94.5 ± 19.18	
Hip, cm	94.41 ± 11.97	95.29 ± 6.30	103 ± 13.52	
SBP, mmHg	115.56 ± 13.36	116.5 ± 10.61	125 ± 7.26	
DBP, mmHg	68.13 ± 6.47	71.53 ± 8.48	75.75 ± 7.81	
**Biochemistry**				
Fasting glucose, mmol/L	5.11 ± 0.30	5.43 ± 0.54	7.94 ± 1.94	0.007
0.5 h glucose, mmol/L	8.66 ± 1.48	9.69 ± 1.16	12.78 ± 3.36	0.007
1 h glucose, mmol/L	8.16 ± 1.59	10.15 ± 1.71	15.32 ± 4.57	< 0.001
2 h glucose, mmol/L	6.86 ± 0.76	8.07 ± 1.62	13.31 ± 4.39	< 0.001
Fasting INS, mIU/L	8.96 ± 5.06	10.4 ± 6.10	20.73 ± 9.31	
0.5 h INS, mIU/L	69.95 ± 37.23	72.65 ± 45.17	50.13 ± 16.62	
1 h INS, mIU/L	81.74 ± 60.94	97.88 ± 65.77	74.25 ± 30.16	
2 h INS, mIU/L	69.05 ± 43.26	106.47 ± 84.83	98.13 ± 52.21	0.035
AUC glucose	22.80 ± 3.0	26.59 ± 2.91	38.73 ± 10.92	
AUC insulin	190.69 ± 117.25	228.96 ± 142.09	183.80 ± 72.85	
HbA1c (%)	5.46 ± 0.14	5.76 ± 0.26	6.85 ± 0.91	< 0.001
HOMA-IR	2.05 ± 1.23	2.55 ± 1.67	7.72 ± 5.39	
HOMA-β	111.87 ± 55.41	113.13 ± 62.21	102.44 ± 49.86	
M	539.48 ± 15.64	580.36 ± 33.33	521.52 ± 53.29	
ISI	66.30 ± 12.77	55.63 ± 17.64	31.25 ± 11.28	0.032
DI	509.81 ± 163.98	440.62 ± 258.20	188.87 ± 187.09	
CRP, mg/L	1.05 ± 1.78	2.10 ± 3.16	5.33 ± 4.08	

Continuous variables with normal distribution or approximately normal distribution were represented as mean ± standard deviation; Categorical variables were presented as numbers (%). The statistically significant difference was defined as P < 0.05. Type 2 diabetes group was excluded from formal comparative statistical analyses; only descriptive statistics were presented for this subgroup.

HC, healthy control; Pre-DM, pre-diabetes; T2DM, type 2 diabetes.

BMI, body mass index; SBP, systolic blood pressure; DBP, diastolic blood pressure; HbA1c, hemoglobin A1c; OGTT, oral glucose tolerance test; 0.5-h, 0.5-hour glucose at OGTT; 1-h, one-hour glucose at OGTT;2-h, two-hour glucose at OGTT; HOMA-IR, Homeostasis model assessment of insulin resistance; M, peripheral glucose uptake rate; ISI, insulin sensitivity index; DI, Disposition index.

### Bacterial community structure (beta diversity) between the HC and Pre-DM groups

**Figure 2:**
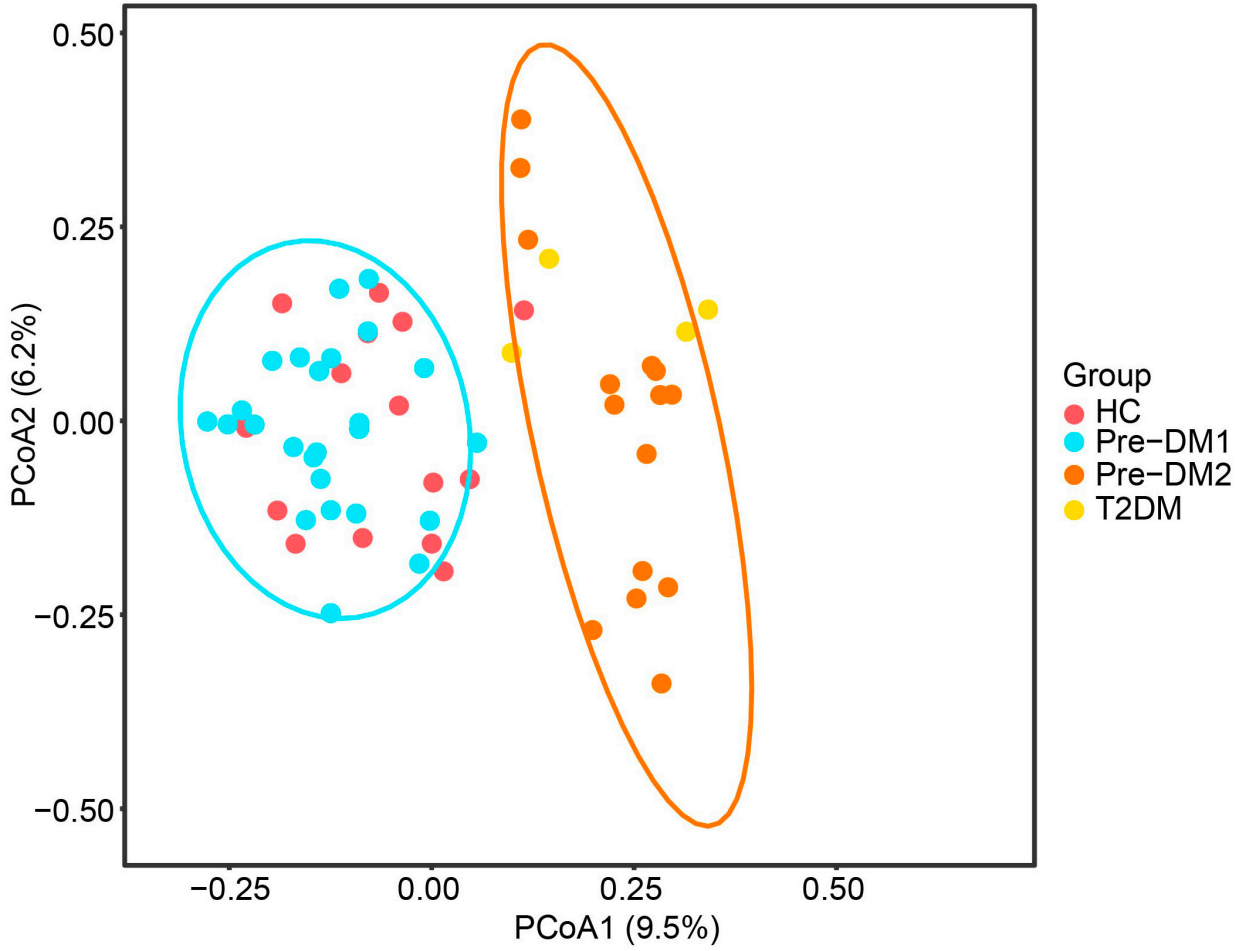
PCoA plot based on Bray Curtis analysis. Dots represent individuals. Type 2 diabetes group was excluded from formal comparative statistical analyses; only descriptive statistics were presented for this subgroup. Pre-DM1 and Pre-DM2 represent different clusters according to PCoA analysis.

A total of 1,390 Operational Taxonomic Units (OTUs) defined at 97% sequence were quantified in this study. To examine the bacterial community structure in the stool samples, a principal coordinate analysis (PCoA) based on Bray-Curtis distances and a permutational MANOVA (pMANOVA) were used for analysis. The gut microbiota of the HC group did not differ significantly from that of Pre-DM (Figure 2). Interestingly, the Pre-DM group exhibited two distinct clustering patterns based on β-diversity analysis: Pre-DM1 (*n* = 25) and Pre-DM2 (*n* = 15). The gut microbiota structure of Pre-DM1 overlapped with the HC group, while Pre-DM2 clustered closer to the type 2 diabetes group. No significant differences were observed in Clinical characteristics between Pre-DM1 and Pre-DM2 groups (Supplementary Table S1). It was worthy to notice that CRP was higher in Pre-DM2 compared with Pre-DM1 (3.11 vs. 1.50 mg/L, *p* = 0.083), although it didn't reach statistical significant.

### Gut microbial community composition in the HC and pre-DM groups

**Figure 3:**
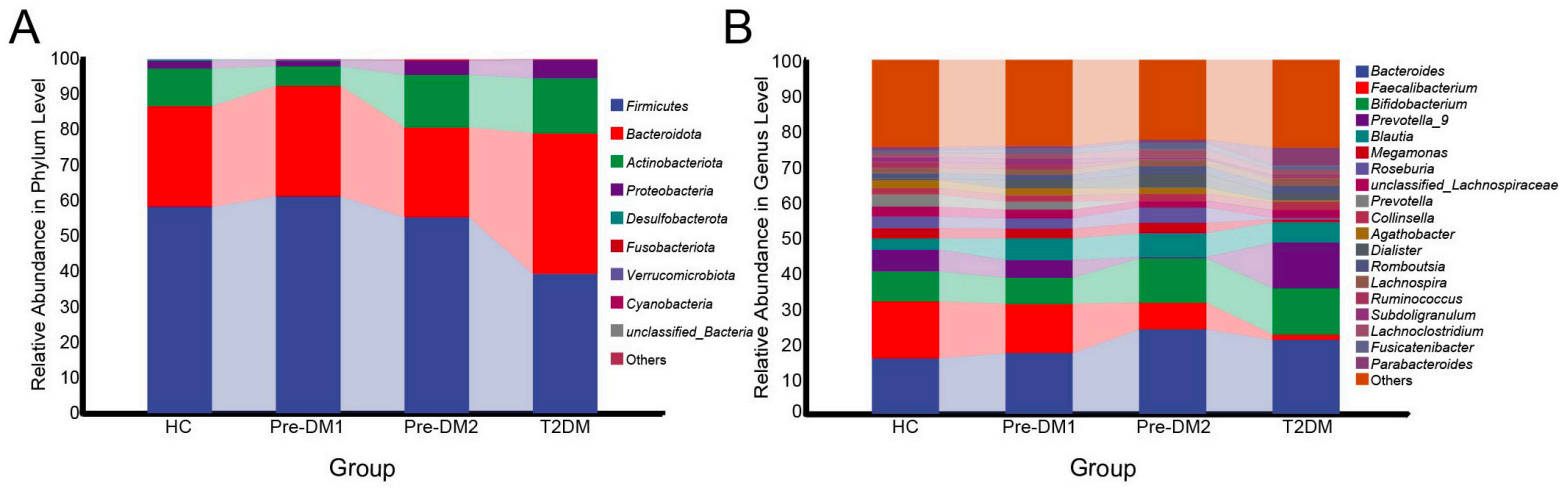
**(A)**Top 10 relative abundance of the gut microbiota at the phylum level between HC, Pre-DM1, Pre-DM2, and T2DM groups. **(B)** Top 20 relative abundance of the gut microbiota at the genus level between HC, Pre-DM1, Pre-DM2, and T2DM group. HC, healthy control; Pre-DM, pre-diabetes; T2DM, type 2 diabetes. Pre-DM1 and Pre-DM2 represent different clusters according to beta diversity.

The composition of the gut microbiota varied among there groups at multiple taxonomic levels. At the phylum level, *Firmicutes, Bacteroidetes*, and *Actinobacteria* were predominant, collectively accounting for over 95% of all sequences in each subject. The relative abundances of *Firmicutes* (*F*) and *Bacteroidota* (*B*) as well as the ratio of *F/B* did not differ significantly among the HC, Pre-DM1, and Pre-DM2 groups. Further, t did not show any significant difference among HC, Pre-DM1, and Pre-DM2 group. Compared to Pre-DM1, *Proteobacteria* was increased in Pre-DM2 (Figure 3A). At the genus level, 19 genera had a relative abundance exceeding 1%. Compared to the HC group, the relative abundances of *Faecalibacterium, Ruminococcus*, and *Subdoligranulum* unchanged in Pre-DM1 group but were significantly reduced in Pre-DM2 group. In addition, while the relative abundance of *Lachnoclostridium* did not differ between HC and Pre-DM1 group. However, compared with the Pre-DM2 group, it was significantly decreased in the Pre-DM1 than in Pre-DM2 (Figure 3B). There were no significant changes in genus composition were observed between HC and combined Pre-DM group (Supplementary Figure S1B).

### Gut microbial alpha diversity in the HC and pre-DM groups

**Figure 4:**
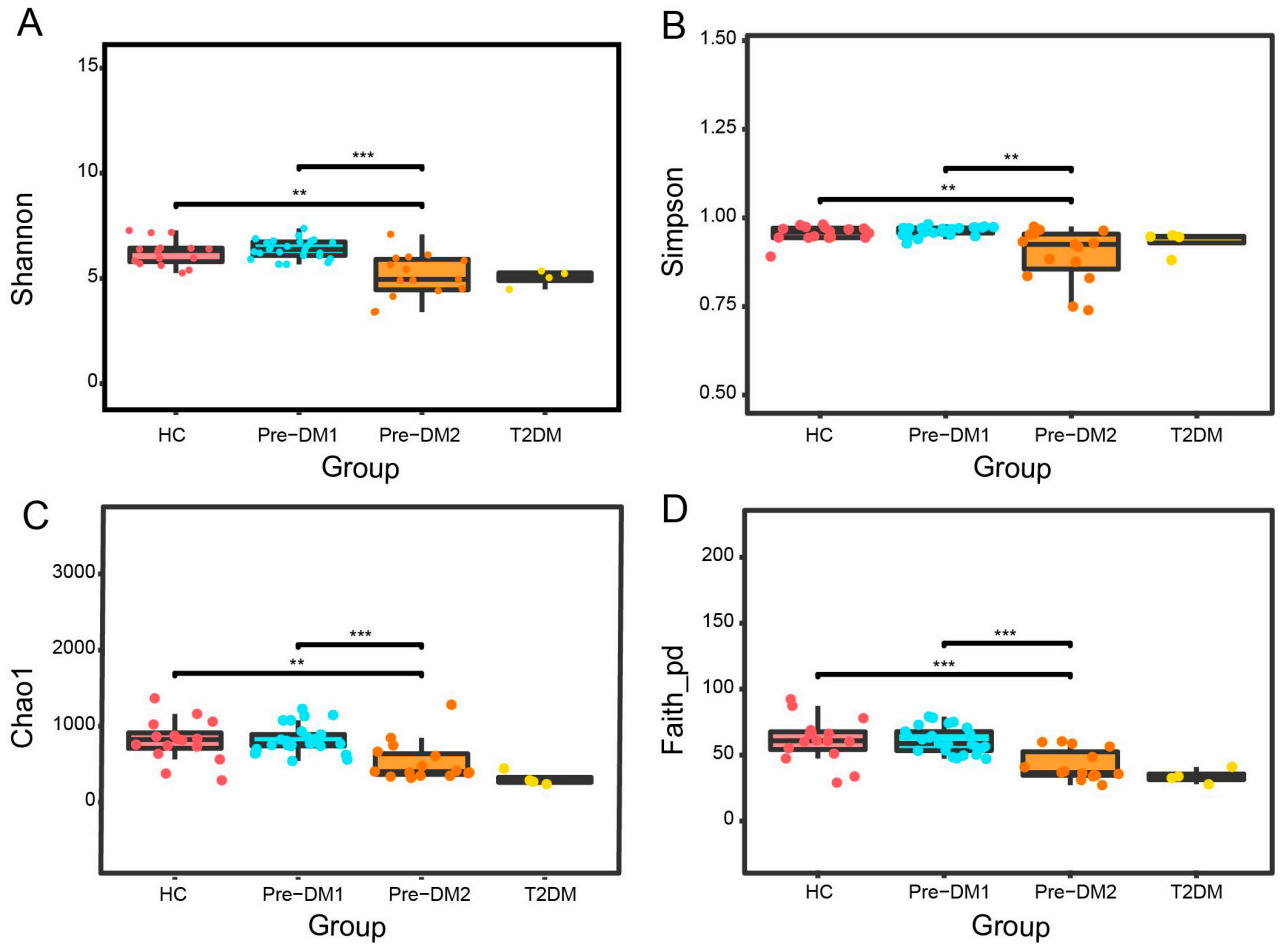
Box plots showing the alpha diversity with significant differences among the HC, Pre-DM1, and Pre-DM2 group. Type 2 diabetes group was excluded from formal comparative statistical analyses; only descriptive statistics were presented for this subgroup. **(A)** Shannon index, **(B)** Simpson index, **(C)** Chao_1 index, and **(D)** Faith's PD index. *p*-value based on the results of One-way ANOVA and in-between groups' comparisons using *post-hoc* LSD test. The statistically significant difference was defined as *P* < 0.05. HC, healthy control; Pre-DM, pre-diabetes; T2DM, type 2 diabetes. Pre-DM1 and Pre-DM2 represent different clusters according to PCoA analysis. **P < 0.01; ***P < 0.001.

The changes in the gut microbiota community structure (α-diversity) of women were evaluated based on microbiota richness (Chao1 index), microbiota diversity (Shannon and Simpson indices) and evolutionary diversity (Faith's PD index) as shown in Figure 4. In summary, microbial richness and diversity of Pre-DM1 group were similar to HC group. In contrast, Microbial richness and diversity in Pre-DM2 group exhibited significant decrease compared with HC group. However, when the Pre-DM1 and Pre-DM2 groups were combined as a whole group, no significant differences in α-diversity were observed relative to HC group (Supplementary Figure S2).

### Gut microbiota signature in the HC and pre-DM groups

**Figure 5:**
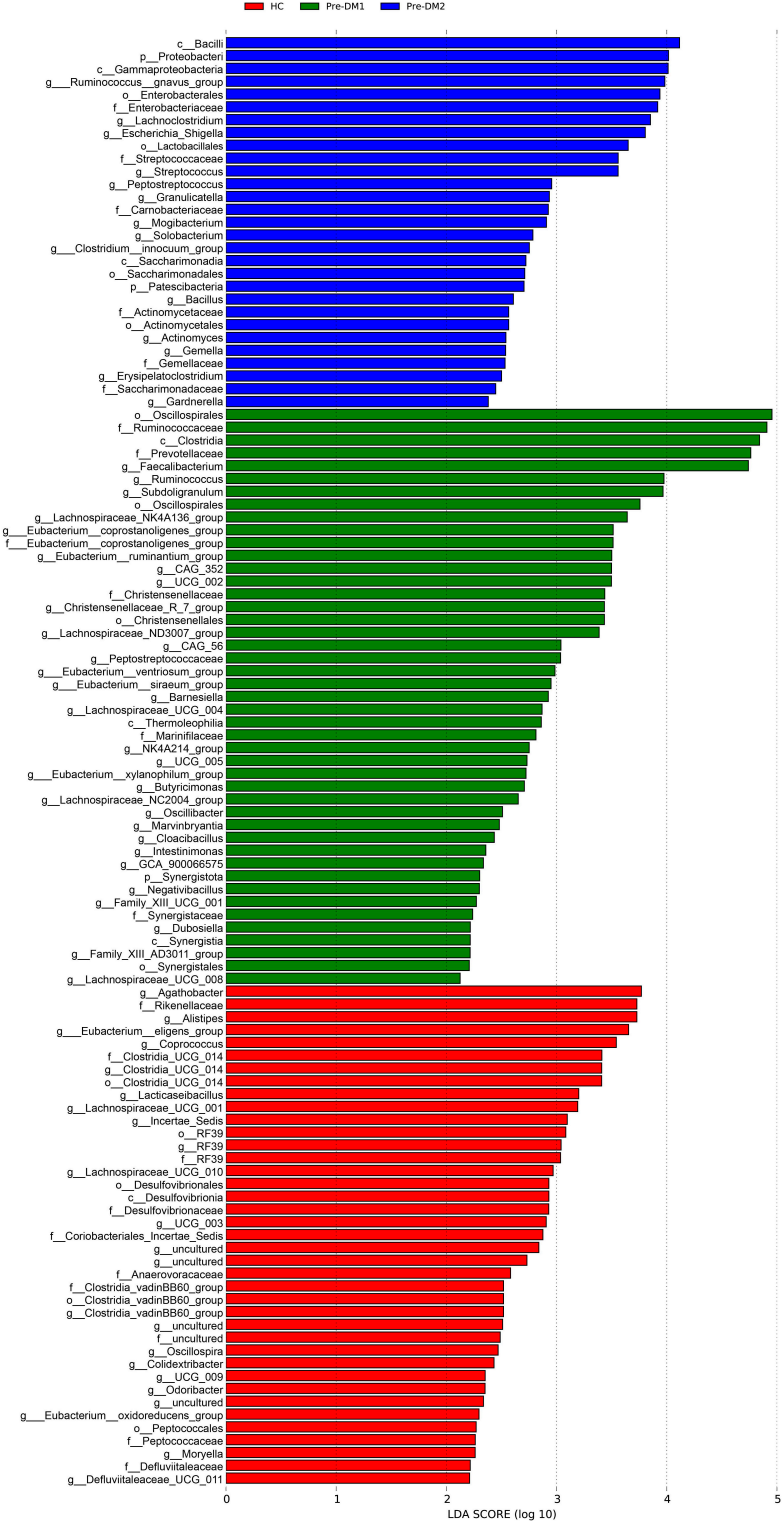
Taxonomic biomarkers identified at the genus level for the three groups using linear discriminant analysis (LDA) with LEfSe. Type 2 diabetes group was excluded from formal comparative statistical analyses; only descriptive statistics were presented for this subgroup. The colors indicated taxa enriched in different groups (red, HC; green, Pre-DM1; blue, Pre-DM2). The LDA score ≥ 2.0 and *p*-value < 0.05 were considered statistically significant. HC, healthy control; Pre-DM, pre-diabetes; T2DM, type 2 diabetes.

To identify taxonomic biomarkers for each group, we performed LEfSe analysis (Figure 5). Genera *Agathobacter*, Alistipes, and *Eubacterium__eligens_group* were identified as biomarkers for the HC group. Genera *Faecalibacterium, Ruminococcus*, and *Subdoligranulum* were biomarkers for the Pre-DM1 group, while genera *Ruminococcus__gnavus_group, Lachnoclostridium*, and *Escherichia_Shigella* were biomarkers for the Pre-DM2 group. To control for the potential confounding effects of age and BMI, we further analyzed the data using MaAsLin2 method. After this adjustment, there was no difference of microbial composition at the genus level between the HC and the Pre-DM1 group. However, compared to HC group, genera Alistipes, *Eubacterium__eligens_group, Faecalibacterium, Ruminococcus*, and *Subdoligranulum were decreased and Ruminococcus__gnavus_group, Lachnoclostridium*, and *Escherichia_Shigella* were increased in the Pre-DM2 (Supplementary Figure S3).

### Fecal SCFA levels in the HC and pre-DM groups

**Figure 6:**
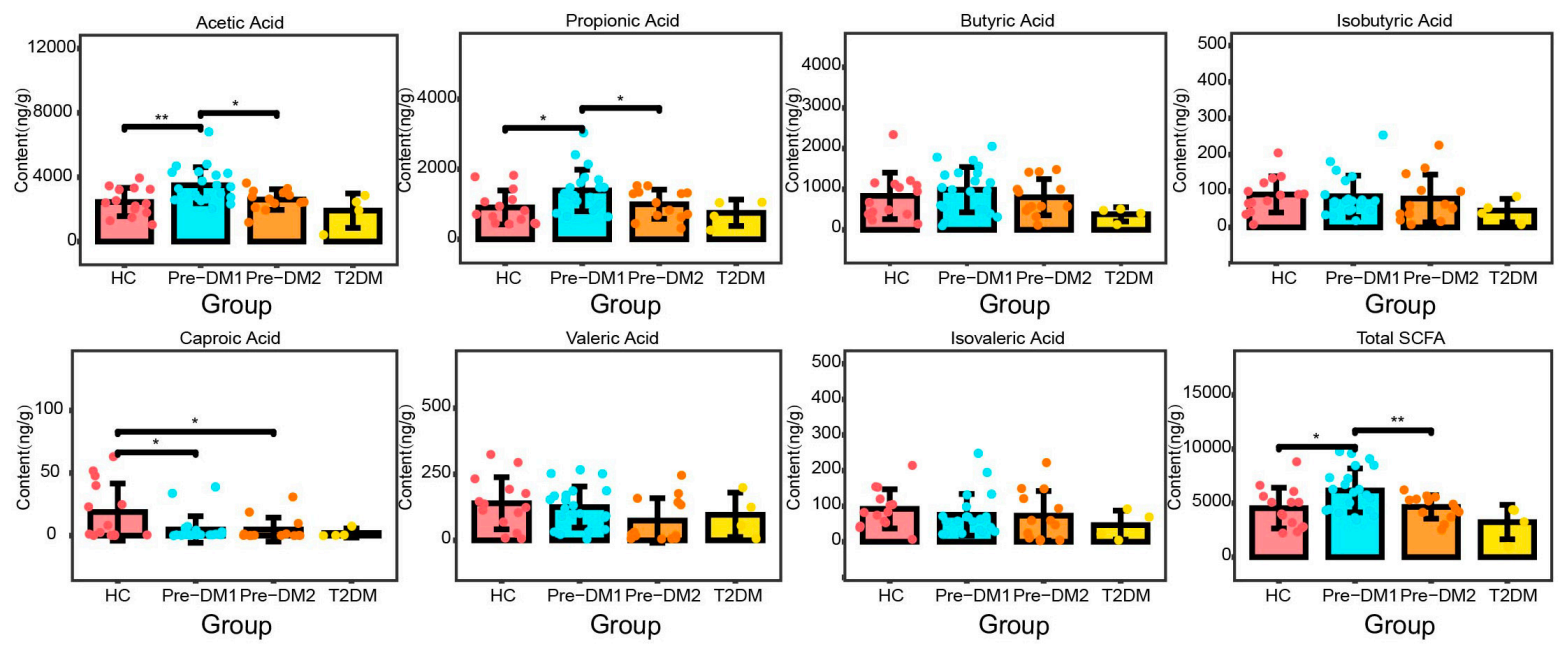
Bar plots showing the abundance of the SCFAs. One-way analysis of variance (ANOVA) was used to compare differences among the HC, Pre-DM1, and Pre-DM2 group, followed by *post-hoc* LSD tests for pairwise comparisons. Type 2 diabetes group was excluded from formal comparative statistical analyses; only descriptive statistics were presented for this subgroup. The statistically significant difference was defined as *P* < 0.05. HC, healthy control; Pre-DM, pre-diabetes; T2DM, type 2 diabetes. *P < 0.05, **P < 0.01.

The concentrations of fecal SCFAs analyzed by GC/MS were shown in Figure 6. Acetic acid, propionic acid, and butyric acid were the major SCFAs. Compared with HC group, acetic acid and propionic acid levels were increased in the Pre-DM1 group but were similar in Pre-DM2 group. When compared to Pre-DM1, acetic acid and propionic acid levels were decreased in the Pre-DM2 group. Interestingly, HC group had higher concentration of caproic acid than Pre-DM1 (18.85 ± 22.83 vs. 4.90 ± 10.67 μg/g, *p* = 0.01), Pre-DM2 (18.85 ± 22.83 vs. 4.85 ± 9.57 μg/g, *p* = 0.02). Meanwhile, when the Pre-DM1 and Pre-DM2 groups were combined, acetic acid levels remained significantly elevated in relative to HC group (Supplementary Figure S4). Moreover, isobutyric acid, isovaleric acid, and valeric acid didn't show any significant differences among groups.

### Correlation analyses among gut microbiota abundance, SCFA levels and clinical indicators

**Figure 7:**
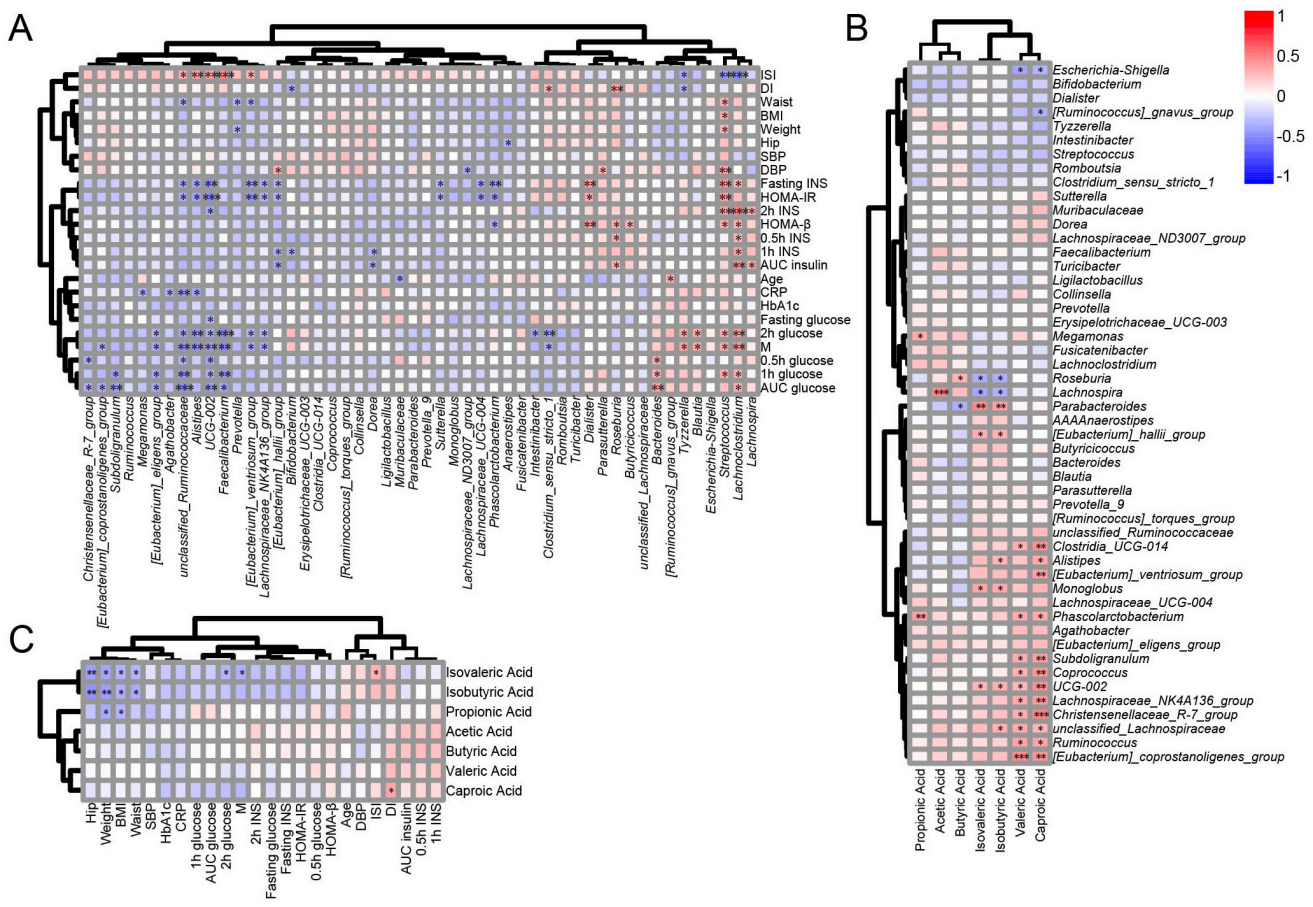
Correlations of bacterial abundances with clinical characteristics **(A)**; correlations of fecal SCFAs with clinical characteristics **(B)**; correlations of bacterial abundances with fecal SCFAs **(C)**. The strength and polarity of correlation was color-coded. The intensity of the color represents the degree of association. The statistically significant difference was defined as *P* < 0.05. *P < 0.05; **P < 0.01; ***P < 0.001.

Spearman correlation was used to identify the associations among gut microbiota abundance, SCFA levels and clinical indicators regardless of glycemic status (Figure 7). *Faecalibacterium* was negatively correlated with 1 h glucose, 2 h glucose, AUC glucose, and M, but positively correlated with ISI. *Subdoligranulum* was negatively correlated with 1 h glucose and AUC glucose. *unclassified_Ruminococcaceae* exhibited negative correlations with 0.5 h glucose, 1 h glucose, 2 h glucose, AUC glucose, fasting insulin, HOMA-IR and M, while showing positive correlation with ISI. Conversely, *Lachnoclostridium* was positively correlated with glucose metabolism and insulin resistance related indicators such as glucose, insulin after glucose loading and negatively correlated with ISI. *Streptococcus* showed a similar correlation pattern to *Lachnoclostridium*. The level of isobutyric acid and isovaleric acid were negatively correlated with BMI, waist circumference, and hip circumference. Caproic Acid level were positively associated with DI. In terms of microbiota-SCFA associations, acetic acid level was positively correlated with the abundance of *Lachnospira*. The level of propionic acid was positively correlated with *Phascolarctobacterium, Megamonas*. The caproic acid level was positively correlated with various genera such as *Subdoligranulum, Ruminococcus, Clostridia_UCG-014, unclassified_Lachnospiraceae* and negatively correlated with *[Ruminococcus]_gnavus_group* and *Escherichia-Shigella*.

## Discussion

This study demonstrated that significant alterations in gut microbiota community structure and fecal SCFA levels were already evident in women with Pre-DM within 1–5 years after a GDM pregnancy. Notably, based on the analysis of microbial β-diversity, Pre-DM group divided into two clusters: Pre-DM1 group which closely resembled that of HC and Pre-DM2 group which was more similar to type 2 diabetes gruop. Alterations of gut microbial composition was more pronounced in women with Pre-DM2 despite comparable age, BMI and months after delivery between Pre-DM1 and Pre-DM2 groups.

Compared with HC, the richness and diversity of the gut microbiota were significantly reduced in Pre-DM2 group. Similar findings were reported in obesity (Le Chatelier et al., 2013; Kubota-Takamori et al., 2025), insulin resistance (Kubota-Takamori et al., 2025), inflammatory bowel disease (Manichanh et al., 2006), cancer (Chen et al., 2017) and even mental disorders (Hsiao et al., 2013), indicating that a decline in richness and diversity is a shared feature across many pathological conditions. Furthermore, the obese individuals with lower bacterial richness gain more weight over time (Le Chatelier et al., 2013). Kelly et al. (2016) including 55 participants with the highest and 57 with the lowest lifetime burdens of cardiovascular disease (CVD) risk factors from Bogalusa Heart Study demonstrated that increased microbial richness was consistently associated with decreased lifetime CVD risk profile. It was worthy to notice that the richness and diversity of the gut microbiota were not significantly different between HC and Pre-DM1 group. The result provided evidence that studies of alterations in gut microbiota might define subsets of Pre-DM and thereby contributed to solve some of the heterogeneity.

*Firmicutes* and *Bacteroidetes* are two major bacterial phyla. Early studies in humans as well as in mice reported that obesity was characterized by an increased *F/B ratio* (Verdam et al., 2013). Furthermore, the abundance of *Firmicutes* increased together with a corresponding decreased in *Bacteroidetes* were found in the spontaneous mouse model of metabolic syndrome (Nishitsuji et al., 2017). However, some other studies on metabolic diseases did not always support these results (Qin et al., 2012; Karlsson et al., 2013; Larsen et al., 2010). A study by Larsen and colleagues involving 18 male type 2 diabetes and 18 healthy male controls demonstrating that the proportion of the phylum *Firmicutes* and the class *Clostridia* were significantly reduced in individuals with type 2 diabetes (Qin et al., 2012). *Firmicutes* was also seen in a low abundance in cohorts of type 2 diabetes patients in a Chinese study (Larsen et al., 2010). Our results showed that the abundances of *Firmicutes* and *Bacteroidetes* and *F/B* ratio were similar among HC, Pre-DM1, and Pre-DM2 groups. Our findings were consistent with previous research on polycystic ovary syndrome (PCOS; Lindheim et al., 2017). Some of the discrepancies between studies can be explained by differences of age, gender, dietary, methods for gut microbiota analysis.

Gut Microbial Community Composition analysis revealed that dysbiosis of the gut microbial structure had occurred even in Pre-DM and further showed the subsets of pre-DM with remarkable heterogeneity. The depletion of *Faecalibacterium*, a butyrate-producing bacterium, in obesity and type 2 diabetes maight impair insulin sensitivity (Salamon et al., 2018; Gao et al., 2018; Zhang et al., 2019). Our data partly supported this finding by showing that the abundance of *Faecalibacterium* was lower in women with Pre-DM2, but not inPre-DM1 group. The ability of *Faecalibacterium* to inhibit the growth and reproduction of pathogenic strains, prevent bacterial translocation and reduce intestinal permeability have been associated with positive effects for the host (Cunningham et al., 2021).

*Ruminococcus* has been shown to be less abundant in individuals with a high cardiovascular disease (CVD) risk profile and associated with a decreased lifetime CVD risk profile from Bogalusa Heart Study (Zhu et al., 2013). *Ruminococcus* was reported to be depleted as well in adults with obesity and type 2 diabetes (Salamon et al., 2018; Gao et al., 2018). Furthermore, a prospective study with a follow-up of 6 years indicated that higher levels of *Ruminococcus* were associated with lower odds of type 2 diabetes (Wang et al., 2024). Our results were much in line with these findings showing that *Ruminococcus* significantly reduced in Pre-DM2 group in a comparison with the HC group. In contrast with these, however, Salamon et al. (2018) and Zhang et al. (2013) found that *Ruminococcus* was more abundant in participants with type 2 diabetes. *Ruminococcus* presented divergent change in the type 2 diabetes group when compared to the healthy controls suggesting that different strains of *Ruminococcus* may be involved. The 16S rRNA gene amplicon sequencing method was unable to investigate this finding at a deeper taxonomic resolution and shot-gun sequencing-based Metagenomics will be needed to address the issue.

The significant decrease in *Lachnoclostridium* abundance in Pre-DM1 group with respect to Pre-DM2 group was accompanied by a significant positive correlation with glucose metabolism and insulin resistance related indicators, while negative correlation with ISI in our study. In a previous study, *Lachnoclostridium* which was a potentially pathogenic bacteria increased the odds of type 2 diabetes, positively associated with circulating metabolites implicated in type 2 diabetes development (Wang et al., 2024). Additionally, in a study of C57BL/6J mice with high-fat diet (HFD)-induced obesity, the abundance of *Lachnoclostridium* was positively correlated with obesity and insulin resistance (Kurakawa et al., 2024). Another butyrate-producing bacterium *Subdoligranulum* did not show any significantly difference in Pre-DM1 group, while significantly reduced in Pre-DM2 group when compared to the HC group. In a Danish study, *Subdoligranulum* was proved to decrease in the type 2 diabetes group and metformin treatment might increase its abundance (Zhang et al., 2013). Therefore, we suppose that *Subdoligranulum* may play a protective effect against hyperglycemia.

SCFAs exert various beneficial health effects including an improved gut barrier function and reduced intestinal inflammation, an important fuel for the colonocytes and modulation of gut hormones release (Blaak et al., 2020). In a Chinese study, the fecal concentrations of acetic acid, propionic acid, and butyrate were all significantly decreased in the type 2 diabetes patients compared with the healthy subjects (Zhao et al., 2019). Another study from United Kingdom proved that increased fecal butyrate level was associated with improved insulin response following an OGTT, whereas propionic acid was causally related to increased risk of type 2 diabetes in normo-glycemic individuals (Sanna et al., 2019). Fecal propionic acid concentration was also higher in the overweight and obese subjects than the lean subjects, while fecal acetic acid and butyrate concentrations were comparable among the three groups (Schwiertz et al., 2010). Our findings showed that acetic acid and propionic acid were increased in the Pre-DM1 group while were similar to Pre-DM2 group when compared to HC group. The discrepancy probably stemed from different stages of glucose metabolism disorder. Caproic acid, a minor SCFAs, was decreased in both Pre-DM1 and Pre-DM2 group relative to the HC group a finding consistent with previous research in adults with type 2 diabetes (Zhao et al., 2019). Moreover, caproic acid was also reported to reduce in Crohn's disease and ulcerative colitis, both of which were associated with severe inflammation (Vich Vila et al., 2023). The mechanisms and functions remained unknown because lack of relevant studies.

The strengths of our research are that young adults with few concomitant medications and comorbidities, well-matched subgroups of subjects and the detailed clinical phenotyping available. The main limitations of our present study are the small sample size and the fact that lifestyle and diet, which may affect both blood glucose levels and the gut microbiota, are impossible to assess in the present study. However, *Post-hoc* power analysis calculation showed that the power analysis was sufficient (>96%) to detect a significant difference among HC, Pre-DM1, and Pre-DM2 group (Cohen's effect size *f* : 0.559–0.807). Additionally, the 16S rRNA gene-based method unable to identify subspecies and function of the bacteria calls for future shotgun-based sequencing studies of gut microbiota.

## Conclusion

Our study suggested that dysregulation of gut microbiota and fecal SCFAs was already in women with Pre-DM after a GDM pregnancy. In particular, we recognized that Pre-DM group divided into two clusters based on β-diversity analysis: Pre-DM1 resembling HC group and Pre-DM2 group with marked alterations. Prospective studies with lager samples are warranted to explore whether Pre-DM2 group with more obvious changes in the gut microbiota is more likely to progress to type 2 diabetes than Pre-DM1.

## Data Availability

The data presented in this study are publicly available. The data can be found at: https://www.ncbi.nlm.nih.gov/, accession PRJNA1299139.
