# Supplementary material for: Association of gut microbiota and short-chain fatty acids with pre-diabetes and diabetes following gestational diabetes
Source: Front Microbiol. 2026 Jan 26;17:1681153. doi: 10.3389/fmicb.2026.1681153 (PMC12883633; doi:10.3389/fmicb.2026.1681153)
Supplement: Supplementary file 1 [file Supplementary_file_1.docx]

Table S1. Characteristics of the study population among the four subgroups

|  | HC  (N = 16) | Pre-DM1  (N = 25) | Pre-DM2  (N=15) | T2DM  (N = 4) | P | | | |
| --- | --- | --- | --- | --- | --- | --- | --- | --- |
|  |  |  |  |  | HC vs  Pre-DM1 | | HC vs  Pre-DM2 | Pre-DM1 vs  Pre-DM2 |
| Descriptive measurements | | | | | |  |  |  |
| Age, years | 36.19±3.8 | 36.56±5.54 | 37.47±4.32 | 34.75±6.08 | |  |  |  |
| High school education and above, n (%) | 14(87.5%) | 21(84.0%) | 12(80%) | 2(50%) | |  |  |  |
| Family history of diabetes, n (%) | 7(43.8%) | 10(40.0%) | 5(33.3%) | 2(50%) | |  |  |  |
| insulin requirement during pregnancy, n (%) | 2(12.5%) | 12(48%) | 7(46.7%) | 3(75%) | |  |  |  |
| months post delivery | 42.19±15.41 | 35.8±14.34 | 39.07±15.86 | 27.50±19.28 | |  |  |  |
| Weight, kg | 61.76±13.37 | 58.74±8.05 | 58.14±11.67 | 71.98±19.95 | |  |  |  |
| BMI, kg/m^2^ | 24.14±4.92 | 23.46±2.85 | 23.65±3.41 | 28.23±6.73 | |  |  |  |
| Waist, cm | 79.57±11.14 | 81.33±5.57 | 83.31±9.51 | 94.5±16.18 | |  |  |  |
| Hip, cm | 94.41±11.97 | 95.73±5.80 | 94.54±7.2 | 103±13.52 | |  |  |  |
| SBP, mmHg | 115.56±13.36 | 116.48±8.85 | 116.53±13.41 | 125±7.26 | |  |  |  |
| DBP, mmHg | 68.13±6.47 | 71.20±5.87 | 72.07±11.85 | 75.75±7.81 | |  |  |  |
| Biochemistry | | | | | |  |  |  |
| Fasting glucose, mmol/L | 5.11±0.30 | 5.42±0.50 | 5.44±0.61 | 7.94±1.94 | | 0.047 |  |  |
| 0.5h glucose, mmol/L | 8.66±1.48 | 9.88±1.15 | 9.38±1.15 | 12.78±3.36 | | 0.004 |  |  |
| 1h glucose, mmol/L | 8.16±1.59 | 10.10±1.70 | 10.24±1.79 | 15.32±4.57 | | 0.001 | 0.001 |  |
| 2h glucose, mmol/L | 6.86±0.76 | 7.83±1.69 | 8.46±1.48 | 13.31±4.39 | | 0.037 | 0.003 |  |
| Fasting INS, mIU/L | 8.96±5.06 | 9.89±4.94 | 11.24±7.79 | 20.73±9.31 | |  |  |  |
| 0.5h INS, mIU/L | 69.95±37.23 | 73.78±47.16 | 70.78±43.21 | 50.13±16.62 | |  |  |  |
| 1h INS, mIU/L | 81.74±60.94 | 93.34±58.74 | 105.43±77.70 | 74.25±30.16 | |  |  |  |
| 2h INS, mIU/L | 69.05±43.26 | 97.16±70.87 | 122.0±105.0 | 98.13±52.21 | |  |  |  |
| AUC glucose | 22.80±3.0 | 26.60±3.05 | 26.57±2.78 | 38.73±10.92 | | <0.001 | 0.001 |  |
| AUC insulin | 190.69±117.25 | 220.65±128.79 | 242.83±165.76 | 183.80±72.85 | |  |  |  |
| HbA1c, % | 5.46±0.14 | 5.80±0.23 | 5.71±0.30 | 6.85±0.91 | | <0.001 | 0.005 |  |
| HOMA-IR | 2.05±1.23 | 2.41±1.29 | 2.77±2.19 | 7.72±5.39 | |  |  |  |
| HOMA-β | 111.87±55.41 | 106.45±53.23 | 124.27±75.57 | 102.44±49.86 | |  |  |  |
| M | 539.48±15.64 | 585.23±31.23 | 572.25±31.23 | 521.52±53.29 | |  | 0.049 |  |
| ISI | 66.30±12.77 | 56.89±16.31 | 53.53±20.08 | 31.25±11.28 | |  | 0.036 |  |
| DI | 509.81±163.98 | 452.23±284.69 | 421.26±214.78 | 188.87±187.09 | |  |  |  |
| CRP, mg/L | 1.05±1.78 | 1.50±1.66 | 3.11±4.62 | 5.33±4.08 | |  | 0.045 | 0.083 |

Continuous variables with normal distribution or approximately normal distribution were represented as mean±standard deviation; Categorical variables were presented as numbers (%). The statistically significant difference was defined as P<0.05. Type 2 diabetes group was excluded from formal comparative statistical analyses; only descriptive statistics were presented for this subgroup.

HC, healthy control; Pre-DM, prediabetes; T2DM, type 2 diabetes.

BMI, body mass index; SBP, systolic blood pressure; DBP, diastolic blood pressure; HbA1c, hemoglobin A1c; OGTT, oral glucose tolerance test; 0.5-h, 0.5-hour glucose at OGTT; 1-h, one-hour glucose at OGTT;2-h, two-hour glucose at OGTT; HOMA-IR, Homeostasis model assessment of insulin resistance; M, peripheral glucose uptake rate; ISI, insulin sensitivity index; DI, Disposition index


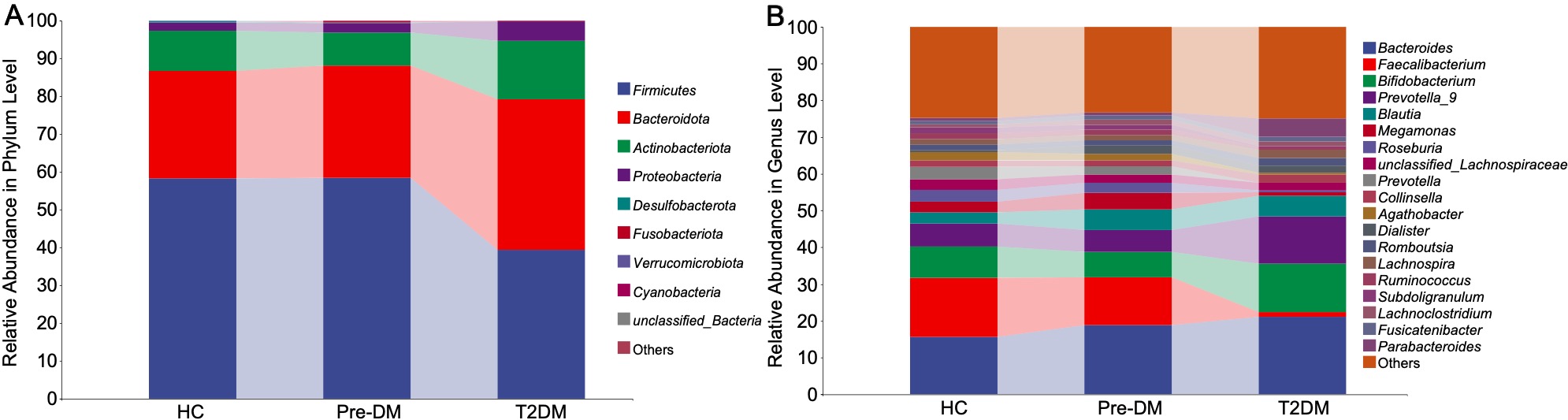


Fig.S1. (Fig.S1A) Top 10 relative abundance of the gut microbiota at the phylum level between HC, Pre-DM and T2DM groups. (Fig.S1B) Top 20 relative abundance of the gut microbiota at the genus level between HC, Pre-DM and T2DM groups.

HC, healthy control; Pre-DM, pre-diabetes; T2DM, type 2 diabetes.

Fig.S2. Box plots showing the alpha diversity with significant differences among the HC and Pre-DM groups. Type 2 diabetes group was excluded from formal comparative statistical analyses; only descriptive statistics were presented for this subgroup. (Fig.S2A) Shannon index, (Fig.S2B) Simpson index, (Fig.S2C) Chao_1 index and (Fig.S2D) Faith’s PD index. The statistically significant difference was defined as P<0.05.

HC, healthy control; Pre-DM, pre-diabetes; T2DM, type 2 diabetes.

Fig.S3. Differentially abundant genera among the HC, Pre-DM1 and pre-DM2 groups using The Microbiome Multivariable Associations with Linear Models (MaAsLin2) method. The statistically significant difference was defined as P<0.05.

HC, healthy control; Pre-DM, pre-diabetes;

Fig.S4. Bar plots showing the abundance of the SCFAs. Comparisons between HC and Pre-DM group were performed using Student’s t-test. Type 2 diabetes group was excluded from formal comparative statistical analyses; only descriptive statistics were presented for this subgroup. The statistically significant difference was defined as P<0.05.

HC, healthy control; Pre-DM, pre-diabetes; T2DM, type 2 diabetes. *P<0.05.
